# A Multi-Institutional Analysis of Prostate Cancer Patients With or Without 68Ga-PSMA PET/CT Prior to Salvage Radiotherapy of the Prostatic Fossa

**DOI:** 10.3389/fonc.2021.723536

**Published:** 2021-10-01

**Authors:** Nina-Sophie Schmidt-Hegemann, Constantinos Zamboglou, Reinhard Thamm, Chukwuka Eze, Simon Kirste, Simon Spohn, Minglun Li, Christian Stief, Christian Bolenz, Wolfgang Schultze-Seemann, Peter Bartenstein, Vikas Prasad, Ute Ganswindt, Anca-Ligia Grosu, Claus Belka, Benjamin Mayer, Thomas Wiegel

**Affiliations:** ^1^ Department of Radiation Oncology, University Hospital, Ludwig-Maximilians Universität (LMU) Munich, Munich, Germany; ^2^ Department of Radiation Oncology, Medical Center – University of Freiburg, Faculty of Medicine, University of Freiburg, Freiburg, Germany; ^3^ German Cancer Consortium Deutsches Konsortium für Translationale Krebsforschung (DKTK), Partner Site Freiburg, Freiburg, Germany; ^4^ Berta-Ottenstein-Programme, Faculty of Medicine, University of Freiburg, Freiburg, Germany; ^5^ Department of Radiation Oncology, University Hospital Ulm, Ulm, Germany; ^6^ Department of Urology, University Hospital, LMU Munich, Munich, Germany; ^7^ Department of Urology, University of Ulm, Ulm, Germany; ^8^ Department of Urology, Medical Center – University of Freiburg, Faculty of Medicine, University of Freiburg, Freiburg, Germany; ^9^ Department of Nuclear Medicine, University Hospital, LMU Munich, Munich, Germany; ^10^ Department of Nuclear Medicine, University of Ulm, Ulm, Germany; ^11^ Department of Therapeutic Radiology and Oncology, Innsbruck Medical University, Innsbruck, Austria; ^12^ German Cancer Consortium (DKTK), Partner Site Munich, Munich, Germany; ^13^ Institute for Epidemiology and Medical Biometry, Ulm University, Ulm, Germany

**Keywords:** prostate, cancer, PSMA PET/CT, biochemical recurrence, radiotherapy

## Abstract

**Introduction:**

68Ga-PSMA PET/CT is associated with unprecedented sensitivity for localization of biochemically recurrent prostate cancer at low PSA levels prior to radiotherapy. Aim of the present analysis is to examine whether patients undergoing postoperative, salvage radiotherapy (sRT) of the prostatic fossa with no known nodal or distant metastases on conventional imaging (CT and/or MRI) and on positron emission tomography/computed tomography (68Ga-PSMA PET/CT) will have an improved biochemical recurrence-free survival (BRFS) compared to patients with no known nodal or distant metastases on conventional imaging only.

**Material and Methods:**

This retrospective analysis is based on 459 patients (95 with and 364 without 68Ga-PSMA PET/CT). BRFS (PSA < post-sRT Nadir + 0.2 ng/ml) was the primary study endpoint. This was first analysed by Kaplan-Meier and uni- and multivariate Cox regression analysis for the entire cohort and then again after matched-pair analysis using tumor stage, Gleason score, PSA at time of sRT and radiation dose as matching parameters.

**Results:**

Median follow-up was 77.5 months for patients without and 33 months for patients with 68Ga-PSMA PET/CT. For the entire cohort, tumor stage (pT2 vs. pT3-4; p= <0.001), Gleason score (GS ≤ 7 vs. GS8-10; p=0.003), pre-sRT PSA (<0.5 vs. ≥0.5ng/ml; p<0.001) and sRT dose (<70 vs. ≥70Gy; p<0.001) were the only factors significantly associated with improved BRFS. This was not seen for the use of 68Ga-PSMA PET/CT prior to sRT (p=0.789). Matched-pair analysis consisted of 95 pairs of PCa patients with or without PET/CT and no significant difference in BRFS based on the use of PET/CT was evident (p=0.884).

**Conclusion:**

This analysis did not show an improvement in BRFS using 68Ga-PSMA PET/CT prior to sRT neither for the entire cohort nor after matched-pair analysis after excluding patients with PET-positive lymph node or distant metastases a priori. As no improved BRFS resulted with implementation of 68Ga-PSMA PET in sRT planning, sRT should not be deferred until the best “diagnostic window” for 68Ga-PSMA PET/CT.

## Introduction

More than half of the men with adverse pathologic features of their prostate cancer will experience biochemical failure, defined by a rise in serum prostate-specific antigen (PSA) level, after radical prostatectomy (RP) (1). In all major guidelines on salvage radiotherapy (sRT) it is advocated that postoperative radiotherapy should be administered at a low level of PSA recurrence (2, 3).

So far, treatment of patients with biochemically recurrent prostate cancer after RP has been guided for years by nomograms to estimate freedom from biochemical failure and distant metastases following postprostatectomy sRT (4). These nomograms demonstrated, that low pre-RT PSA, low Gleason score 6-7, positive surgical margins and high PSA doubling time >10 months are associated with the highest progression-free probability with a known superiority of early sRT at lower PSA levels compared to all other mentioned parameters (4).

Advances in novel positron emission tomography (PET) radiotracers for prostate cancer, above all ^68^Gallium-labeled ligands of the prostate-specific membrane antigen (68Ga-PSMA) are associated with unprecedented sensitivity for localization of biochemically recurrent prostate cancer at low PSA levels as shown by several meta-analyses of retrospective studies (5) and lately by a prospective multicentre trial including 635 patients (6). Consequently, 68Ga-PSMA PET/CT has a high impact on the management of biochemically recurrent prostate cancer as assessed by several retrospective and prospective analyses leading to changes in treatment in more than half of patients with biochemical recurrence (7, 8). Hypothetically, 68Ga-PSMA PET/CT’s high impact and subsequently individualization of treatment could possibly translate into improved biochemical recurrence free and ultimately overall survival. This has been analysed so far by a few studies mostly without a comparator group of patients treated without prior 68Ga-PSMA PET/CT (9, 10).

Currently, a Phase III trial (NCT03582774) explicitly analysing the oncologic benefit of an additional 68Ga-PSMA PET/CT prior to sRT is underway with the aim to prove that the incorporation of 68Ga-PSMA PET/CT in sRT planning will improve 5-year BRFS by 20% (11). With the results of this trial not to be expected within the next few years, a matched pair analysis of patients with and without 68Ga-PSMA PET/CT prior to sRT of the prostate fossa was undertaken. The aim of this matched pair analysis was to examine whether patients undergoing sRT of the prostate fossa with no known nodal or distant metastases on conventional imaging (CT and/or MRI) and on 68Ga-PSMA PET will have an improved biochemical recurrence-free survival compared to patients with no known nodal or distant metastases on conventional imaging only.

## Material and Methods

### Patient Population

From 1998 - 2017, a total of 672 consecutive patients were referred for sRT after RP due to persistent or rising PSA at the Radiation Oncology departments of four university hospitals. Patients with pathologic lymph nodes at time of RP, distant or lymph node metastases in 68Ga-PSMA PET, androgen deprivation therapy (ADT) before or simultaneously with sRT, prior history of RT or incomplete documentation were excluded. All patients received sRT of the prostatic bed only. Thus, the following analysis is based on 459 patients. Of this cohort, 364/459 (79%) patients were treated without a 68Ga-PSMA PET and 95/459 (21%) received a 68Ga-PSMA PET/CT prior to sRT. This retrospective analysis was performed in compliance with the principles of the Declaration of Helsinki and its subsequent amendments (12) and was approved by the local Ethics Committee of the respective medical university centers. The requirement to obtain informed consent was waived.

### Statistical Analysis

Biochemical recurrence-free survival (BRFS), defined as PSA < post-radiotherapy Nadir + 0.2 ng/ml from the last day of sRT, was the primary outcome. The effect of 68Ga-PSMA PET/CT and other important clinical parameters on BRFS was first analysed by means of Kaplan-Meier analysis using the log-rank test as well as by uni- and multivariable Cox regression analyses for the entire cohort. Multivariable Cox-regression analysis was used to identify predictors of BRFS after sRT. The effect of 68Ga-PSMA PET/CT on BRFS was then additionally assessed after a propensity score (PS) matching (1:1 ratio) has been conducted using tumor stage (pT2 *vs.* pT3-4), Gleason score (GS ≤ 7 *vs.* GS8-10), PSA at time of sRT (<0.5 *vs.* ≥0.5 ng/ml) and radiation dose (<70 *vs.* ≥70 Gy) as matching variables. The PS was calculated using a logistic regression model and the final matching was done using the calculated PS as a measure of distance within an optimal matching approach (13). Differences in BRFS after the PS-matching were assessed by means of a Cox proportional hazards model using a robust sandwich covariance matrix estimator to account for the clustered structure introduced by the PS-matching. Differences between subgroups were compared using Mann-Whitney-U, Student’s t- and Chi-square test with a p-value of <0.05 considered statistically significant.

## Results

### Patients’ Characteristics and Outcome for the Entire Cohort

Patients had primarily pT2 prostate cancer (52% of the pre-68Ga-PSMA PET patients and 61% of patients with 68Ga-PSMA PET). Patient cohorts differed significantly regarding Gleason score and surgical margins with a higher percentage of 68Ga-PSMA PET patients with a Gleason Score ≥ 7 (93% *vs.* 64%; p<0.001) and surgically negative resection margins (69% *vs.* 47%; p<0.001). Further, 68Ga-PSMA PET-patients had a significantly higher median pre-SRT PSA levels (0.33 ng/ml *vs.* 0.29 ng/ml; p<0.007) compared to patients of the pre-68Ga-PSMA PET era. Median follow-up was 77.5 months (range 0-157) for patients without and 33 months (range 3-63) for patients with 68Ga-PSMA PET/CT. Thirty-one patients (33%) had evidence of PET-positive local recurrence. Patients’ characteristics are listed in **Table 1**.

**Table 1:** Patient characteristics.

Factor	no PSMA-PET/CTN=364	PSMA-PET/CTN=95	p-value
Year of RP Median FU (range)	1989 - 2015 77.5 months (0 - 157)	2000 - 2017 33 months (3 - 63)	<0.001*
iPSA ng/ml Mean (SD) Median (IQR)	12.2 (10.0) 9.2 (6.2 - 14.3)	12.8 (12.2) 10.2 (6.0 - 14.4)	0.814*
Tumor stage pT2 pT3-4	190 (52%) 174 (48%)	58 (61%) 37 (39%)	0.123**
Gleason score GS ≤6 GS 7 GS 8-10	132 (36%) 160 (44%) 72 (20%)	7 (7%) 63 (67%) 25 (26%)	<0.001**
Surgical margins R0 R1 Rx	172 (47%) 160 (44%) 32 (9%)	65 (69%) 26 (27%) 4 (4%)	<0.001**
Post-RP PSA nadir <0.1 ng/ml ≥0.1 ng/ml	288 (79%) 76 (21%)	75 (79%) 20 (21%)	0.970**
Time between surgery and PSA recurrence	12 (0-149)	25 (0-137)	<0.001*
Pre-SRT PSA ng/ml Mean (SD) Median (IQR)	0.52 (0.84) 0.29 (0.15 - 0.51)	0.54 (0.67) 0.33 (0.23 - 0.51)	0.007 *
SRT dose Gy Mean (SD) Median (IQR)	69.3 (2.6) 70.2 (66.6 – 72.0)	69.2 (3.0) 70.2 (66.0 – 72.0)	0.796***

*Mann-Whitney-U test; **Chi-square test; ***Student’s t-test.

RP, radical prostatectomy; iPSA, initial PSA; GS, Gleason Score; SRT, salvage radiotherapy; SD, standard deviation; IQR, inter quartile range.

**Figure 1:**
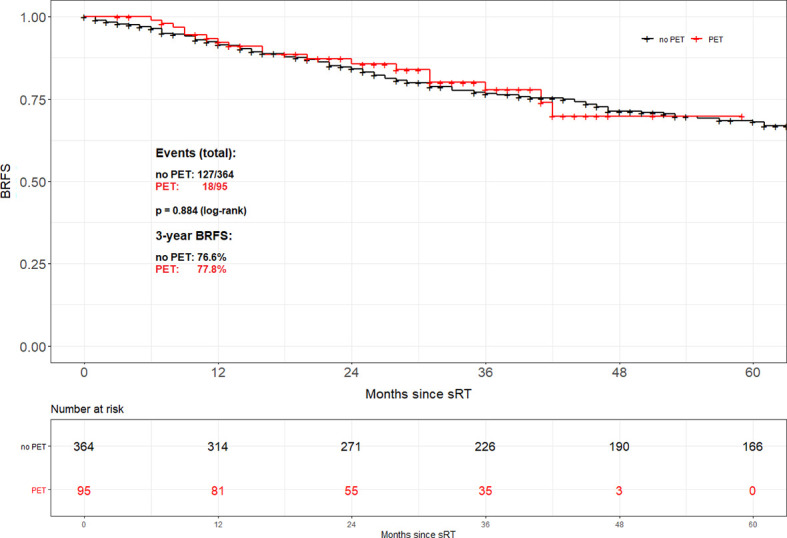
Biochemical recurrence-free survival according to the use of PSMA PET/CT for the entire cohort.

For the entire cohort, no difference in BRFS (**Figure 1**) depending on the use of 68Ga-PSMA PET was observable (2-year BRFS 84.1% for non-PET-group *vs.* 85.6% for PET-group and 3-year BRFS 76.6% *vs.* 77.8%, p=0.884, respectively). A multivariable cox regression analysis (**Table 2**) was conducted to assess whether there was an association between tumour or treatment specific variables and BRFS. Overall, tumor stage (pT2 *vs.* pT3-4; p<0.001), Gleason score (GS ≤ 7 *vs.* GS8-10; p=0.003), PSA at time of sRT (<0.5 *vs.* ≥0.5ng/ml; p<0.001) and radiation dose (<70 *vs.* ≥70Gy; p<0.001) were the only factors significantly associated with BRFS. No significant association was observed for the use of 68Ga-PSMA PET/CT prior to sRT (p=0.789), initial PSA (<10 ng/ml *vs.* ≥ 10 ng/ml; p=0.508), surgical margins (R0 *vs.* R1; p=0.055) and post-prostatectomy PSA (<0.1 ng/ml *vs.* ≥ 0.1 ng/ml; p=0.192).

**Table 2:** Multivariable Cox Regression Analysis on factors associated with biochemical recurrence free survival after SRT.

Factor	HR (95% CI)	p
PSMA-PET/CT (no*/yes)	1.07 (0.64 - 1.79)	0.789
iPSA 10 (<10*/≥10 ng/ml)	1.13 (0.79 - 1.60)	0.508
Tumor stage (pT2*/pT3-4)	2.29 (1.60 - 3.27)	<0.001
Gleason score (GS ≤ 7*/GS8-10)	1.77 (1.22 - 2.57)	0.003
Surgical margins (R0*/R1)	0.72 (0.52 - 1.01)	0.055
Post-RP PSA nadir (<0.1*/≥0.1 ng/ml)	1.28 (0.88 - 1.87)	0.192
Pre-SRT PSA (<0.5*/≥0.5 ng/ml)	2.00 (1.39 - 2.86)	<0.001
SRT dose (<70*/≥70 Gy)	0.54 (0.39 - 0.76)	<0.001

*State of reference.

HR, Hazard Ratio; CI, confidence interval; iPSA, initial PSA; GS, Gleason Score; RPE, radical prostatectomy; SRT, salvage radiotherapy.

### Patient Characteristics and Outcome After Propensity Score Matching

**Figure 2:**
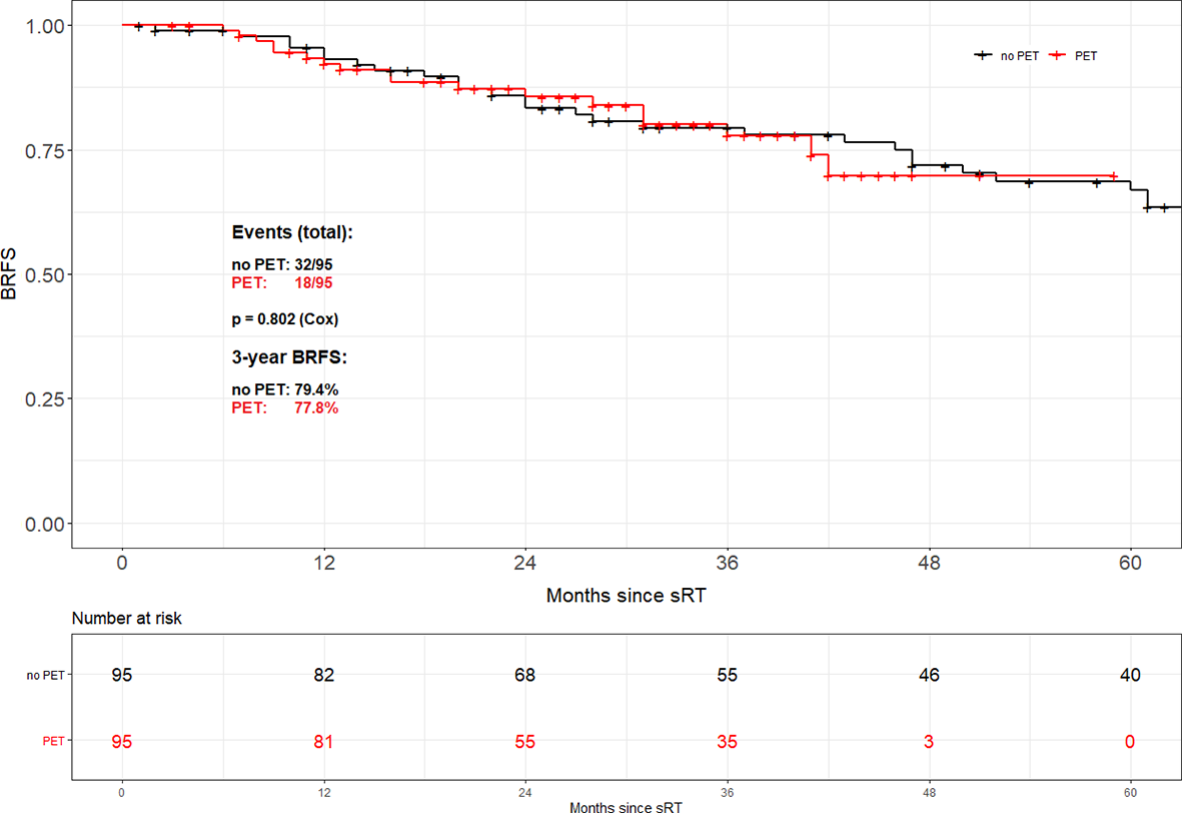
Biochemical recurrence-free survival according to the use of PSMA PET/CT after propensity score matching.

**Figure 3:**
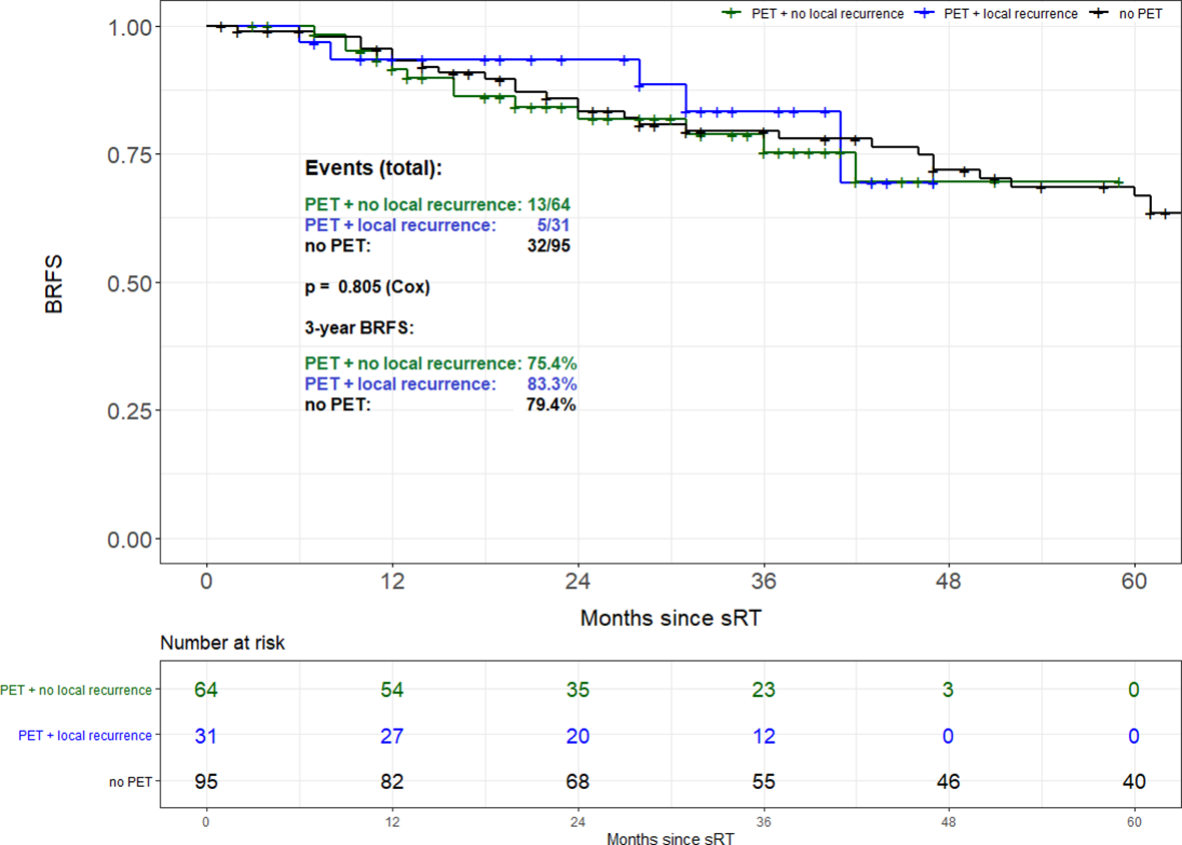
Biochemical recurrence-free survival of patients with PET-positive local recurrence vs. patients without PET/CT.

Propensity score matching based on tumor stage (pT2 *vs.* pT3-4), Gleason score (GS ≤ 7 *vs.* GS8-10), PSA at time of sRT (<0.5 *vs.* ≥0.5 ng/ml) and radiation dose (<70 *vs.* ≥70 Gy) resulted in 95 patient pairs. Assessment of both the area of common support of the PS distributions in PET and no PET patient groups as well as the absolute standardized difference (ASD) after the matching was done revealed perfectly balanced comparison groups. The common support area nearly reached 100% overlapping, and ASD values for all variables in the PS model were <0.1. Consequently, there was almost no pair of case and control patient which differed in any value of all the matching variables. Overall, no difference in BRFS based on the use of 68Ga-PSMA PET/CT prior to sRT (3-year BRFS 77.8% *vs.* 79.4%; p=0.802) could be found (**Figure 2**). Equally no difference in BRFS was evident when comparing patients with PET-positive local recurrences within the prostatic fossa to patients without PET/CT prior to sRT or a negative PET/CT (p=0.805) (**Figure 3**). Patients with PET-positive local recurrence had significantly higher median pre-sRT PSA values compared to PET-negative patients and patients without a PET/CT (0.46ng/ml *vs.* 0.29 ng/ml *vs.* 0.24ng/ml, p= 0.001).

## Discussion

The introduction of 68Ga-PSMA PET/CT imaging has substantially improved the detection and localization of macroscopic disease in patients with biochemical recurrence after RP. 68Ga-PSMA PET/CT allows for an individualization of treatment in terms of irradiation volumes, applied overall dose and concomitant ADT (14–16). This has led to a surge in the use of 68Ga-PSMA PET/CT particularly across Europe compared to the United States, where 68Ga-PSMA PET has been approved by the US Food and Drug Administration for institutional use at the University of California, Los Angeles (UCLA) and the University of California, San Francisco (UCSF). Only recently, a phase III trial corroborated the high detection rates of 68Ga-PSMA PET/CT at low PSA levels in patients with biochemical recurrence ranging from 38% for a PSA level <0.5 ng/ml to 57% for 0.5 to <1.0 ng/ml (6). Consequently, the European guidelines on prostate cancer cautiously recommend to perform a PSMA PET/CT post-prostatectomy at PSA levels >0.2 ng/ml (3).

To assess the oncologic benefit of 68Ga-PSMA PET/CT in patients with or without a PET-positive local recurrence within the prostatic fossa and with prior exclusion of patients with PET-positive lymph node or distant metastases, a matched pair analysis of patients treated with sRT of the prostate fossa without *vs.* patients with 68Ga-PSMA PET/CT prior to sRT was undertaken. For the entire cohort, no significant difference in BRFS between patients with or without 68Ga-PSMA PET/CT was observed, although the two cohorts differed significantly with more adverse features in the 68Ga-PSMA PET cohort, namely higher Gleason score, higher pre-sRT PSA and higher percentage of patients with R0-resection being present. Subsequently, not 68Ga-PSMA PET/CT but pre-sRT PSA (<0.5 *vs.* ≥0.5 ng/ml), tumor stage (pT2 *vs.* pT3-4), Gleason score (GS ≤ 7 *vs.* GS8-10), and radiation dose (<70 *vs.* ≥70 Gy) were the only factors significantly associated with BRFS. Several retrospective studies have affirmed the prognostic role of the pre-sRT PSA level with a potential chance of cure in more than 60% of patients treated before PSA rises >0.5ng/ml (4, 17, 18). Likewise, the association of dose-escalation in the sRT setting with relapse-free survival was previously confirmed in multiple retrospective analyses with the oncologic results of the SAKK 09/10, a phase III trial on the potential benefit of dose-escalation still pending (19, 20).

After matching according to these factors with an overall 95 pairs of patients again no difference in BRFS was evident, nor was a significant difference in BRFS seen when comparing patients with a PET-positive local recurrence to patients without 68Ga-PSMA PET.

Once again, this underlines the significant influence of pre-sRT PSA on BRFS after sRT with higher PSA-levels correlating with macroscopic local and/or lymph node recurrences and diminished BRFS rates. Thus, based on these findings in a selective cohort of patients with exclusion of patients with 68Ga-PSMA PET-positive lymph node or distant metastases a priori, the current analysis supports the recommendations by several guidelines on prostate cancer that PSMA PET/CT should be performed in patients with PSA >0.2 ng/ml and sRT should not be postponed until a PSMA PET-positive result is observed (3, 21).

This is especially true as in contrast to the pre-PSMA PET era, when the 3 major studies on adjuvant radiotherapy were initially published (1, 22, 23) a certain reluctance can nowadays be observed among urologists but as well radiation oncologists to perform adjuvant radiotherapy in men with adverse pathologic features. This tendency most likely stems from an increase in RT-associated side effects e.g. urinary incontinence or erectile dysfunction when applying early postoperative RT in comparison to sRT (24). In addition, no difference in 5-year BRFS and even 8-year metastasis-free or overall survival was observed in retrospective studies initiating sRT at low PSA levels (25, 26).

A further increase of sRT will most likely be observed based on the latest results of the three randomised studies RADICALS-RT, RAVES and GETUG-AFU 17 all comparing adjuvant radiotherapy to a policy of early sRT triggered at low PSA failures of maximum 0.2ng/ml after RP (27). All three only recently published studies indicate the possibility of an observation policy with sRT after RP as long as sRT is initiated at low PSA levels (28–30).

With a known better outcome for patients receiving early sRT at PSA levels ≤ 0.5 ng/ml (4), the fundamental maxim of sRT might as such be “the earlier, the better” (4). In particular, Bartkowiak et al. advocate for very early sRT at PSA levels of 0.2 ng/ml or less (18) with a known risk for further metastases at a PSA level of 0.4 ng/ml and rising (31). The significance of an early sRT start at low PSA-levels is further depicted in the work by Shelan et al. showing that even dose-escalated sRT with short-course ADT in patients with macroscopic local recurrences after RP leads to inferior tumor control compared to early sRT (32).

Thus, not surprisingly, the present data reveal that not the availability of a 68Ga-PSMA PET/CT is decisive for BRFS after sRT but the initiation of sRT at low PSA-levels with patients treated without a PSMA PET having significantly lower PSA levels prior to sRT. This underlines the dilemma of modern imaging with 68Ga-PSMA PET/CT, which so far has a superior detection of relapses than any other imaging modality for prostate cancer but is still not sensitive enough for the low PSA levels associated with the highest chance of long-term BRFS after sRT. Nevertheless, with growing body of evidence PSMA PET will maintain its dominant role in staging patients at initial diagnosis before curative-intent surgery or radiotherapy, as seen in proPSMA trial, at the time of postoperative PSA relapse as well as in the treatment setting of metastatic castration-resistant prostate cancer patients who do receive [(177)Lu]-PSMA-617 radionuclide treatment (6, 33, 34).

The present study has several limitations mainly due to its retrospective nature. Based on varying institutional policies, the treatment protocols and the follow-up procedure were not identical for all patients. The influence of 68Ga-PSMA PET might therefore be disguised by the comparably high overall median dose in the sRT setting of 70.2 Gy in both cohorts. For the cohort of patients without 68Ga-PSMA PET/CT the precise staging method (CT and/or MRI) was not known for each patient. A further shortcoming of the present analysis that precludes drawing final conclusions is the relatively short follow-up of patients with 68Ga-PSMA PET/CT. We tried to overcome these issues by performing a matched-pair analysis with a reasonably high number of 95 patient pairs for statistical analyses. To avoid further biases, patients with ADT were excluded resulting in a BRFS free of the influence of ADT.

## Conclusion

This multi-institutional analysis did neither confirm an improvement in BRFS for the entire cohort nor after matched-pair analysis nor for patients with PET-positive local recurrences using 68Ga-PSMA PET/CT prior to sRT compared to a pre-PSMA PET cohort after excluding patients with PET-positive lymph node or distant metastases a priori. Overall, the significance of a low PSA before the initiation of sRT was reconfirmed in the present analysis. As no improved BRFS resulted with implementation of 68Ga-PSMA in sRT planning, sRT should not be deferred until the best “diagnostic window” for PSMA PET/CT. Further advances in PSMA PET/CT like the recent emergence of Fluorine-18 tracers with promising detection rates of 61.5% for patients with PSA values as low as 0.2 - 0.5 ng/ml might further influence BRFS rates post-sRT (35).

## Data Availability Statement

The raw data supporting the conclusions of this article will be made available by the authors, without undue reservation.

## Ethics Statement

The studies involving human participants were reviewed and approved by approval number of the University of Ulm (391/15), approval number of the University of Freiburg (519/17), approval number of the University of Munich (17-765)]. Written informed consent for participation was not required for this study in accordance with the national legislation and the institutional requirements.

## Author Contributions

All authors contributed to the study conception and design. Material preparation, data collection and analysis were performed by N-SS-H, CZ, BM, and TW. The first draft of the manuscript was written by N-SS-H, CZ, BM, and TW. All authors commented on previous versions of the manuscript. All authors contributed to the article and approved the submitted version.

## Conflict of Interest

The authors declare that the research was conducted in the absence of any commercial or financial relationships that could be construed as a potential conflict of interest

## Publisher’s Note

All claims expressed in this article are solely those of the authors and do not necessarily represent those of their affiliated organizations, or those of the publisher, the editors and the reviewers. Any product that may be evaluated in this article, or claim that may be made by its manufacturer, is not guaranteed or endorsed by the publisher.
